# Challenges of palliative care integration into routine hospital care in Iran: A Q-method inquiry on key healthcare providers’ prospects

**DOI:** 10.34172/hpp.43513

**Published:** 2024-12-30

**Authors:** Seemin Dashti, Hassan Mahmoodi, Abdolreza Shaghaghi

**Affiliations:** ^1^Health Education and Promotion Department, Faculty of Health, Tabriz University of Medical Sciences, Tabriz, Iran; ^2^Department of Nursing, Islamic Azad University (IAU), Ardabil Branch, Ardabil, Iran; ^3^Vice Chancellor Office for Public Health Affairs, Kurdistan University of Medical Sciences, Sanandaj, Iran

**Keywords:** End-of-life care, Hospice care, Palliative care, Delivery of healthcare, Healthcare providers, Healthcare system, Hospital-patient relations, Iran

## Abstract

**Background:**

Inclusion of palliative care (PalC) in the routine provided healthcare of hospitals is emphasized by the World Health Organization (WHO) in the endorsed Health Promoting Hospitals (HPH) initiative. Nonetheless, an evidence gap exists about explicit barriers and operational complexities that might prevent embeddedness of PlaC in the Iranian National Healthcare System (INHS) and this was the main impetus for the conception of the current research.

**Methods:**

The Barry and Proops’ recommended Q method procedures were used in 6 phases including concourse development through the scientific literature search and consultation with the 27 key informants, statements’ selection, population set (P-set) selection, Q sorting and factor analysis. Principal component analysis and Varimax rotation were used in factor analysis and the values of factor loadings≥0.4 were considered satisfactory in assessing the degree to which a certain Q sort conforms to a particular factor.

**Results:**

The extracted four factors that accounted for 47% of the total observed variance were shortage of physical space and number of the healthcare providers (HCPs), inadequate involvement of the patient’s family members in end-of-life treatment decisions, communication barriers, and inadequate training of HCPs for PalC provision.

**Conclusion:**

This study elicited important barriers of incorporating PalC into the routine hospital care and hence, importance of taking a multifaceted approach for achieving the goals of INHS in quality healthcare provision. Contrasting views of the approached HCPs could help development of the evidence-based national policies concordant with the HPH initiative in Iran.

## Introduction

 Patients with life-limiting diseases are generally hospitalized in their end-stage phase of illness and experience a range of physical, emotional and psychological challenges that pose profound impacts on their quality of life. Palliative care (PalC) is a set of targeted healthcare that not only could alleviate the patients and their next of kin’s physical, mental and spiritual sufferings but could enhance quality of life in one of their staggering stages of life. PalC as a conspicuous element of health services therefore, is highly crucial when curative and maintenance care are no longer favorable.^1-3^

 Hospital settings are considered as one of the routine and convenient venues for PalC provision worldwide^4,5^ therefore, they need to be prepared and disciplined for accomplishment of the task that is not the case in many parts of the globe. PalC if integrated and provided by trained health care HCPs in the hospitals’ routine healthcare packages is expected to yield a positive impact on the healthcare clients’ (HCCs’) perceived quality of life and meeting one of their salient needs.^6^ The readiness for integration and provision of PalC should be in vain in terms of physical space, human and fiscal resources.

 Inclusion of PalC in the routine hospital services is emphasized by the World Health Organization (WHO) in the endorsed Health Promoting Hospitals (HPH) initiative^6^ which is in cognizant with the recommended action of health services’ reorientation in the Ottawa charter for health promotion.^7^ The HPH concept is referring to hospitals that proactively take action to become a type of organizations that define active and participatory roles for patients and all HCPs in securing high quality medical and nursing services and developing a health promoting organizational structure.^6^ Shared responsibility of healthcare systems in expanding their roles beyond solely providing clinical and curative services and refocusing on the total needs of the individuals were highlighted in the Ottawa charter.^7^ The attention represents importance of having complementary approaches for organizational change and adopting coordinated actions to ensure setting up of a healthcare system in countries that contribute to the pursuit of equitable health in all walks of life.^7,8^ Structuring of equal opportunities to secure optimal quality of life for patients and their family members in hospitals is inextricably aligning with the overall guiding principles in the Ottawa charter that call for embracement of an expanded mandate by healthcare systems to support the needs of individuals for a healthier life.^7,9^

 Even though, many healthcare systems across the world are encountering challenges in responding adequately to the needs of end-stage patients and their family members.^10^ Factors such as paucity of fiscal and non-fiscal resources, inapposite configuration of physical space or intangible infrastructure were suggested to prevent fulfilment of the obligation in accordance with the evidence-informed policy mandates.^10^

 Integration of PalC into the hospitals’ conventional healthcare packages was proclaimed in the conveyed National Accreditation Guideline of the Hospitals (NAGoH) by the Iranian Ministry of Health and Medical Education (MOHME). The recommendation was made to ensure incorporation of best practice standards into the procedural workflow of the teaching and non-teaching hospitals.^11,12^ However, due to variety of organizational constraints such as overwhelming burden of responsibilities, overall shortage of skilled manpower and funding resources and most importantly, a general disharmony and deficit technical knowledge among policy makers about importance of PalC in optimizing quality of life and achieving better health outcomes, an organized plan of action was not laid out to indicate acceptance of responsibility in responding to the unmet healthcare needs of a sizable number of the Iranian patients and their family members.^13^ PalC provision in the country thence, do not have a firm base at the organizational mission and action plan and instead is restricted to self-initiated and internally-triggered attempts by the healthcare staffs who might not even be trained for PalC delivery. Therefore, varying degrees of unmet healthcare needs exist for patients and their family members in the country’s hospitals that require thorough diligence. Challenges ahead of integrating PalC into the hospitals services, span across a myriad of multilayered factors. However, lack of an implicit sense of agency (belief that baseline potential requisites are exist to act something positive) seems to be focal.^14^

 The noticed evidence gap that exists about explicit overarching list of barriers and array of organizational and operational complexities which might prevent embeddedness of PalC^15,16^ in the routine expectrum of hospital-based healthcare services in Iran was the main impetus for the conception of the current research. Therefore, findings from an extensive review of empirical scientific evidence and a groups of knowledgeable frontline Iranian healthcare providers (HCPs) in selected hospitals were consulted to bridge the knowledge gap. The findings might provide a new insight to improve sense of accountability and liability in responding to one of the important unmet healthcare needs of the HCCs in their critical stage of life.

## Materials and Methods

###  Q methodology

 Q methodology was suggested to be a substantiate paradigm for the investigation of human subjectivity.^17^ The method was used in this research to identify different patterns of thought about major impediments of integrating PalC services into the routine hospital care in the INHS.

###  Implementing Q methodology

 The Barry and Proops’^18^recommended Q method procedures in 6 phases were employed in this study as follow:

*1st and 2nd steps* (concourse development)^19^: A concourse space was created through searching and enumerating the reported barriers to hospital-based PalC provision in the scientific literature and consultation with 27 key informants who were selected for concourse purposefully and non-probably. The approached individuals were healthcare professionals working in the Imam Khomeini, Alavi, and Fatemi hospitals of the city of Ardabil, Northwest of Iran who were knowledgeable about PalC due to their educational background and experience.^20^ They were asked to express their opinions about the actual barriers of integrating PalC into the country’s provided routine hospital services in face to face semi-structured interviews. The raised list of barriers in the interviews were added to the list of identified hurdles from the literature search and a non-redundant set of statements were formed.

*3rd step* (screening and selection of statements)^21^: The expert panel consultation (first hand source for concourse statements) yielded 327 statements and after removing redundancies 303 statements remained for further scrutiny. Scoping review of the relevant scientific evidence (second-hand source) brought about 24 additional statements. Precise assessment of these 327 clusters of viewpoints and elimination of irrelevant, repetitive and indefinite statements resulted to emanation of 44 standpoints (Q statements) about main barriers of integrating PalC into routine hospital care packages. These retrieved statements were entered into a tailor-made computer program that had been uniquely adapted for Q-statements’ sorting.

*4th step* (population-set (P-set) selection): The expert panel members who were consulted in the concourse development stage re-contacted for invitation to participate in the Q sorting phase of the analysis. From the 27 purposively invited informants only 24 consented to participate at this stage. The enthusiasts consisted nurses, nursing superintendents, supervisors, matrons, hospitals’ quality improvement officers, social workers, patient education officers, clinical psychologist, oncology and neurology specialists, and assistant nurses working in the selected teaching hospitals.

*5th step* (Q-sorting)^19^: The recruited key informants were asked at this phase to sort the Q statements primarily on a simple three-columns spreadsheet (that were labeled in accord with of a Likert type scaling format: i.e. I strongly agree, I have no opinion, and I strongly disagree) to organize their mind sets and facilitate more efficient sorting of the statements in a later stage. The expert panel members were asked in a next step to resort the classified Q statements on a computer generated quasinormal distribution Q table that was designed in the form of a nine-options Likert-type scaling method from completely agree (4 + ) to completely disagree (-4). At either end of the Q table the most and least important columns were imputed with smallest number of spaces for Q statements since, it is unlikely for the participants to have fierce standpoints about numerous statements.^22^ The informants were requested to rank all Q statements in the provided Q table based on their broadest and most preferred viewpoints and intimate information. Nevertheless, this sorting procedure is necessarily considered to be dependent on the respondents’ subjectivity and therefore, to vary from one person to another.^18,23^ Those participants who classified the statements at the two ends of response options ( + 4 and -4) were also asked to explain their reasons for choosing the extreme answers if they want.

*6th step* (factor analysis and interpretation): This final stage of Q method analysis is quantitative in nature to identify clusters of participants who have indicated a set of shared viewpoints with regard to the Q statements’ sorting pattern.^22^ The Q factor analysis was performed using the PQ-Method software (version 2.35) and applying principal component analysis procedure with Varimax rotation and Eigenvalue > 1. The analysis output was identification of a set of latent factors that were ascertained by the study participants’ composite Q sorting. The values of factor loadings (between −1 and + 1) were pinpointed to assess the degree to which a certain Q sort conforms to a particular factor and the value of equal or greater than 0.4 was considered as the cutoff point.^24^ The estimated eigenvalues and explained variance of each factor were employed to determine the most suitable factor solution. The identified factors with their corresponding Q sorts were discussed with the participant informants to ensure the rigor and reliability of the interpretation.

## Results

 The mean age of the study attendants was 40.33 years and 77.77% of them were female. The mean time for Q statement sorting was 25 minutes. The performed factor analysis extracted four latent factors that accounted for 47% of the total variance. The applied rotation matrix revealed that 13 participants significantly loaded on the first factor, 7 respondents on the second factor, 5 people on the third factor, and 4 informants on the fourth factor. The factor arrays were computed to form a Q table for each factor and scoring each Q statement. The Q statements were ranked in order of the importance of each factor (Table 1).

 The observed small and non-significant values of correlation coefficients between the extracted factors (Table 2) revealed independence of each factor from other factors that could hinder integration of PalC services into the routine hospital care packages in Iran.

**Table 1:** Rank values of the loaded Q statements in the constructed factor arrays and representation of the main barriers of integrating palliative care into the routine hospital care in Iran with significantly different scores on the extracted factors

**Item**	**Statements**	**Factor 1**	**Factor 2**	**Factor 3**	**Factor 4**
		**Rank values**			
1	Limited number of health providers compared with the number of patients.	4*	4*	1	3
2	Discrimination in the provision of care services due to the social status of the patient and family.	-4	3**	-4	0**
3	High defined workload for health providers of some hospital wards.	4**	3	1	3
4	High workload of nurses due to the writing responsibilities.	3*	2	2	1
5	Restrictions for visiting or being companion for the patients relatives.	-3**	2	-4**	1
6	Not identifying the responsible person in the wards who provide PalC to the patient and family members or train health providers.	2	-1	0	2
7	Insufficient training of PalC to health providers.	2	-1	-1	3
8	Lack of a plan to accurately evaluate the quality of provided PalC.	2	-4**	-2	1
9	Impossibility of identifying or categorizing patients who are in need of PalC due to the uncertainty of diagnosis or health status.	0	1**	-1	-1
10	Lack of specific guidelines to explain the importance of PalC and clarify the duties of each health service provider.	2**	-4	-2*	-3
11	Non-concordance of guidelines with existing conditions of hospitals.	3**	0*	3**	1
12	Physical environment limitations to provide the privacy, counseling, bereavement support and other supportive care.	3	0*	3	1
13	Breakdown of, low quality or small number of hospital equipment, including beds and ventilation system, cause a decrease in patient comfort and quality of PalC.	1	2	0	3
14	Inadequate attention by health care providers to all aspects of PalC.	0	-3**	-1	2
15	Negative attitudes of health providers towards PalC, needs of patient/family, and being responsible for them.	-3	-2	-2	0
16	lack of proper cooperation of health providers in providing PalC to the patient/family	-3	0	-2	-1
17	Health providers concern about the impact of bad news on the patient/family.	1	-2	4**	-4**
18	The large number of relatives and companions of the patients, which hinders effective communication and education for them.	-1	0	4**	-3
19	Lack, complications and insufficiency of palliative drugs, which leads to lack of proper control of physical symptoms in patients.	1	0	-2	-4**
20	Due to the lack of PalC team, the hospitals cannot benefit in a processual way from expertise of the relevant specialists, including pain fellowship, psychologist, chaplain, social worker, etc.	3	0**	-3	-3
21	Salaries, benefits, and incentives for health care providers dealing with EOL patients are not commensurate with their workload.	1**	-2	-3	-1
22	Mistrust, false hope or despair of the patient/family members due to receive the contradictory information form health providers about the disease or treatment.	-1	0	2**	0
23	Family members’ disagreement in deciding to choose or not to choose aggressive and unnecessary treatments.	-1	4**	-3**	-2
24	The possibility of the worsening of the patient's condition due to the instability of the patient's hemodynamic status following the implementation of palliative measures such as changing the position or transferring the patient to home according to the family's wishes.	-1	1	0	-2
25	Insufficient financial support for low-income patient/family to continue receiving palliative services.	-2*	3**	-3	-2
26	Putting EOL patients in lower priority than other patients to receive health services.	-3*	-1	0	0
27	patient/family`s denial or resistance to acceptance of incurability disease.	-1	3	2	-2*
28	Poor educability of the patient/family members to receive training and cooperation with health providers.	-1	0*	3**	-1
29	Attitude, believes, feelings and mental conditions of the patient/family members that effect participation in treatment decisions and cooperation with health providers.	0	1	3	0
30	High expectations of family members regarding the patient's recovery process or economic support.	0	1	0	-1
31	Inadequate information of the patient and his family members about the duties of the health providers or the care provided.	1	2	1	1
32	Inadequate attention of family members to the patient and insufficient emotional relationship between them.	-2	2	0*	2
33	The opposition of family members towards breaking bad medical news to the patient.	1	1	2	2
34	Contradiction of the preferences of the patient/family members with moral, legal, or therapeutic principles.	0	0	0	1
35	Lack of interest in health care providers from palliative care.	-2	-3**	0	1
36	Emotional stress of health providers or family members to stop futile and harmful treatments that may lead to the patient death.	0	1	-1	2
37	Lack of financial budget to integrate PalC services into health care packages.	2*	-3*	-1	0
38	Lack of access to the required technologies, such as electronic documentation.	-2	-1	2**	-2
39	Non-acceptance of EOL care rules in clinical wards due to difficulties in coordinating PalC measures with ward work procedures.	-1	-1	1	-1
40	Organizational pressures and policies to speed up the process of discharge of patients lead to lose the opportunity of talking with the patient about his decision regarding resuscitation and intubation in the last days of his life.	-4	-2	-1	0
41	Patient and hospital culture (focus on the patient saving and deny the death).	0	-1	1	-1
42	Poor cooperation of the hospitals wards in providing EOL care.	0	1*	-1	4
43	Time constraints due to the short time interval between hospitalization and death of the patient.	-2	-1	1	0
44	Poor skill and experience of health providers about PalC and patients' needs.	1	-3**	3**	4**

* Significance at *P* < 0.05; ** Significance at *P* < 0.01).

**Table 2:** The estimated level of correlation between the extracted factors referring to the main barriers of integrating palliative care into the routine hospital care in Iran

**Factors**	**Between factors’ correlation coefficients (** ***P*** values *)			
	**1**	**2**	**3**	**4**
Shortage of physical space and number of the HCPs	1	-	-	-
Inadequate involvement of the patient's family members in making treatment decisions in end-of-life (EOL) situations	0.023 (0.879)	1	-	-
Communication barriers between HCPs, patients and their family members	0.149 (0.334)	0.087 (0.574)	1	-
Inadequate training of the HCPs for skillful provision of PalC	0.164 (0.287)	0.110 (0.475)	0.066 (0.668)	1

* *P* values greater than 0.05 is considered to be statistically non-significant (independence of each factor from other factors).

###  Factor 1: Shortage of physical space and number of the HCPs 

 Thirteen participants significantly loaded on this factor that explained 16% of the observed variance. The participants were in believe that limited number of HCPs compared to the admitted number of patients (4 + ), high workload of the HCPs especially in some hospital wards (4 + + ), nurses’ unprecedented paperwork responsibilities (3 + ), delineation of non-concordant clinical guidelines compared to the de-facto conditions of the hospitals’ wards (3 + + ) and inadequacies of physical spaces in terms of HCCs’ privacy and quality provision of counseling, bereavement support and other imperative care (3) are the main barriers in front of PalC integration into the current healthcare packages in the hospitals.

###  Factor 2: Inadequate involvement of the patient’s family members in making treatment decisions in end-of-life (EOL) situations 

 Seven respondents significantly loaded on this factor that explained 11% of the discerned variance. According to this group of informants’ perspectives family members disagreement in deciding to accept or refuse aggressive or what they call unnecessary treatments (4 + + ) for their patients, insufficient financial support for low-income patient/family to receive palliative services (3 + + ) from private sector, patient/family`s denial or resistance to accept incurability of their patient’s illness (3), insufficient number of HCPs in comparison to the number of admitted patients (4 + ), high workload of HCPs especially in some hospital wards (3) are the major barriers a head of PalC incorporation into the hospitals’ provided health services.

###  Factor 3: Communication barriers between HCPs, patients and their family members 

 Five health providers loaded on the third factor that accounted for 9% of the total variance. The panelists referred to concerns and anxiousness of the HCPs about impact of giving bad news on the patient or their family members (4 + + ), presence of a high number of patients’ next of kin in the hospitals’ ward which hinders effective communication and education for them (4 + + ), hassles related to educating and attracting cooperation of the patient/family members with low literacy levels (3 + + ), patient/family members’ psycho-emotional circumstances, attitudes, believes and feelings that might pose influence on their readiness for participation in treatment decisions or cooperation with HCPs (3) and poor skill and experience of HCPs for PalC provision and efficacious assessment of the patients’ needs (3 + + ) as the paramount impediments in front of PalC addition into the list of ongoing hospital services in Iran.

###  Factor 4: Inadequate training of the HCPs for skillful provision of PalC

 Four study participants loaded on this factor that attributed to the 7% of the total observed variance. Based on these experts’ opinions HCPs’ poor skills or experience for competent provision of PalC (4 + + ), reluctance of hospitals’ managers in adding financially demanding services into their already overstretching financial resources (4), insufficient, malfunctioning and faulty hospitals appliances including beds and ventilation system that could interfere with quality PalC provision (3), lack of efficient scheduled training about PalC to HCPs (3) and unbearable workload of the HCPs in some hospital wards (3) are the most consequential barriers in the hospitals for functional PalC provision.

## Discussion

 This study was conducted to identify main impediments to integrate PalC into the routine hospitals’ care in Iran. The applied Q-method inquiry on the key informants’ prospects about the prevailing barriers in the country’s hospitals emanated four latent factors. The recognized hindrances were inadequacies of physical space and number of the HCPs, inadequate involvement of the patients’ family members in making treatment decisions in EOL situations, communication barriers between HCPs, patients and their family members along with inadequate training of the HCPs for skillful provision of PalC.

 Physical space incongruity with PalC provision is a widespread limitation worldwide especially in the resource limited countries and addressed in other studies.^25-27^Shortage of competent HCPs to provide PalC was also pinpointed as a considerable limitation in the literature for decent provision of PalC.^28,29^ The excessive workload of healthcare professionals in hospitals has been identified as a factor that limits their ability to effectively interact with patients’ family members, thereby hindering their capacity to provide suitable emotional and spiritual support.^30,31^ Non-concordant promulgated standard operating procedures with the current workloads in the hospitals’ wards were suggested by the study informants to prevent preservation of the structural integrity that is needed for quality and efficient care provision. The quandary should be investigated separately for the benefit of HCPs and their clients in the hospitals.

 Inadequate involvement of the patient’s family members in making treatment decisions in EOL situations was the other important reported obstacle in front of commensurable PalC provision in the hospitals. Background learned cognition of people in societies about their level and nature of involvement in clinical decision making was stated to be a strong mediating factor in their acceptance and actual engagement in PalC delivery to the patients.^32^ A degree of hesitancy from active cooperation with HCPs in clinical decision making or PalC delivery that stems from low level of health literacy among family members of EOL patients was propagated in some study reports^25^ which needs to be further clarified in future research.

 Other predisposing factor that could potentially infuriate such a reluctance in patients’ next of kin against their active involvement in PalC delivery was suggested to rise from their disagreement about the type and essence of cares that the patients need to receive. Occurrence of conflict between patients’ family members and care teams was revealed before in the critical care units (ICUs).^33^

 Presence of communication barriers between HCPs, patients and their family members was recognized in this study as the third consequential limit that inhibit complete inclusion of PalC in the current provided cares in the country’s hospitals. Impact of the poor literacy level and psycho-emotional conditions of the patients’ next of kin which hamper their potential preparedness and interest for possible cooperation with HCPs were recognized as baseline interference for having effective communication episodes in this study. Poor health communication skills of the HCPs and lack of thorough confidence in their abilities in giving bad news to the EOL patients’ next of kin were identified as other hindering factors of PalC integration into the hospitals’ care packages. This is while, having a good level of communication skill is a sine qua non element for auspicious PalC provision efforts.^34^ Gaps in the HCPs competencies in having assertive and empathic communication with patients and their family members in situations of serious illness and EOL care were also reported in other studies.^35^ Factors such as different spoken language, cultural background and religious beliefs by the same token were suggested to escalate the communication challenges between HCPs and their customers.^25^ However, having a justifiable level of communication skill and developing a trustful and liable therapeutic relationship with the HCCs were implied to be consequential for conducive PalC delivery.^36,37^ Failure of the hospitals’ administration authorities in restriction of the number of patients’ companions in the wards was prompted by the respondents as a prohibiting componentfor a fruitful PalC provision. Such an impediment was also reported in other implemented studies.^38^

 Inadequate training of the HCPs for skillful provision of PalC was another recognized barrier for integration and coordination of the PalC delivery in the Iranian hospitals. The hindrance was indicated to be widespread and important in several countries.^27,39^ To fill the gap, revision of the current taught curriculum of the nursing and other healthcare professionals who may work with EOL patients is vitally important and a prime requisite for their professional paths toward a tenure-responsive approach in healthcare provision.^40,41^ The amendment as a priority action area is in accord with the endorsed reorientation of health services in the Ottawa Charter for health promotion for creating a firm base to support execution of health promoting initiatives in hospitals.^42^ Education of the general population about importance of PalC in provision of a sound and responsive healthcare according to the respondents’ prospect could be a new addition to the healthcare armamentarium especially in resource limited countries that might already be overburdened and fragile due to high workload and demand on the health services.^36,43^

 Inclusion of PalC in the routine provided healthcare services in hospitals is mandated by the WHO in the advocated HPH initiative.^6^ This study results indicated certain inconsistencies between the avowed strategy for health promotion in the country’s hospital settings and proposed plan of cares. The findings shed light on the ambiguities of the health professionals with regard to possible challenges of incorporating PalC into routine hospital cares and actions that can be taken within the current administrative boundaries of the INHS.

## Limitations of the study

 The study results open new avenues for further research on main focus of steps that need to be taken for successful incorporation of PalC into the routine hospital-based health services in Iran. However, the findings warrant to be interpreted by caution due the following methodological limitations. Sampling bias is a prevalent draw back in Q-method studies.^44^ The study participants were recruited purposefully from the representative informants working in the hospitals of only one city (Ardabil) in Iran and while all efforts were made to optimize the respondents’ heterogeneity for attaining the greatest diversity of viewpoints but due to logistic restraints recruitment of key informants working from the country’s other cities and provinces was not feasible.^45^ Therefore, the study findings might not be generalizable to all HCPs in Iran. The identified barriers in this study have been generated through the informants’ responses to the Q-sets and it is highly possible that different factors would be extracted if other statements had been included both from the conducted literature review or consultation of the panelists.^46^ The captured viewpoints therefore, might not reflect the breadth of existent knowledge about the studied phenomenon. The study participants had not same level of knowledge about importance and requisites of PalC provision in hospital settings. The discordant has potential to pose important implications on the expressed standpoints of the respondents.

## Conclusion

 This study elicited important barriers of incorporating PalC into the routine hospital care in Iran. The applied Q method in this study contributed to a broader understanding of the components and dynamics that hinders integration of PalC in the country’s national health system. The study findings suggest that a multifaceted approach must be taken to develop an effective action plan for achieving the goals of INHS in quality healthcare provision. The procured information about the barriers that might prevent successful integration of PalC into the routine hospital care packages could assist decision makers in all the organizational layers to have firm evidence-based alternatives rather than anecdotal choices in disposing the organization’s missions.

## Implications

 PalC provision is regarded as a sine qua non element of standard healthcare packages in several countries of the world as endorsed by the WHO.^6^ The study results provide an array of multi-tiered intervention approaches that are likely to promote organizational functioning and social responsibleness of the INHS. The findings also offer insights for alleviating the obstacles that might hinder efforts to meet HCCs expectations and to ensure successful healthcare outcomes across the world.

## Future direction

 Technical feasibility of the changes that could potentially be considered for the organizational effectiveness improvement and a better healthcare quality in the INHS is still need to be further explored. Compatibility of the solicited ameliorations that are sought to meet the HCCs’ basic healthcare rights with the existing organizations circumstances must also be investigated thoroughly. The current divide between the status quo conditions in the INHS and a requisitioned standard healthcare package should be re-conceptualized in terms of the global ethical mandate that propose for an equitable healthcare provision irrespective of patients’ stage of illness or life. Embeddedness of the fundamental human rights preservation recommendations in the healthcare decision-making processes or even in the ongoing practices within the INHS’s organizational ecosystem is also need an explicit speculation. Planning and implementation of further qualitative or quantitative research to shed light on all these uncertainties might have an utmost significance for achieving the objectives that highlighted in the Ottawa charter.

## Competing Interests

 All the authors declare that they have no known competing financial interests or personal relationships in preparation of the submitted work that could have appeared to impact on its integrity or scientific merit.

## Ethical Approval

 The study was approved by the regional Medical Ethics Board of Trustees (MEBoT) affiliated to the Tabriz University of Medical Sciences (approval number: IR.TBZMED.REC.1400.322). All the study participants have been provided with the study details including the aims and objectives and their rights in leaving the study without obligation to explain the reasons. Those who gave their verbal consent to participate in the study were provided a printed or electronic copy of an informed consent form attached to the study brochure to read and sign.
